# Gene expression profile and response to trastuzumab–docetaxel-based treatment in breast carcinoma

**DOI:** 10.1038/sj.bjc.6605310

**Published:** 2009-09-15

**Authors:** F Végran, R Boidot, B Coudert, P Fumoleau, L Arnould, J Garnier, S Causeret, J Fraise, D Dembélé, S Lizard-Nacol

**Affiliations:** 1 Molecular Genetics Unit, Centre Georges François Leclerc, IFR-Santé-STIC, Dijon Cedex, France; 2 Department of Oncology, Centre Georges François Leclerc, IFR-Santé-STIC, Dijon Cedex, France; 3 Laboratory of Pathology, Centre Georges François Leclerc, IFR-Santé-STIC, Dijon Cedex, France; 4 Roche Laboratory, Neuilly, France; 5 Department of Surgery, Illkirch, France; 6 Institute of Genetics, Molecular and Cellular Biology, Illkirch, France

**Keywords:** HER2-positive breast cancer, trastuzumab, microarray analyses, resistance, predictive signature

## Abstract

**Background:**

Resistance to trastuzumab is often observed in women with human epidermal growth factor receptor 2 (HER2)-positive breast cancer and has been shown to involve multiple potential mechanisms. We examined the ability of microarray analyses to determine the potential markers of pathological complete response (pCR).

**Methods:**

We conducted an analysis of tumours from 38 patients with locally advanced HER2-positive breast cancer who had received trastuzumab combined with docetaxel. Quantitative reverse transcriptase (RT)–PCR was used to assess the expression of 30 key genes; microarray analyses were carried out on 25 tumours to identify a prognostic gene expression profile, with 13 blinded samples used to validate the identified profile.

**Results:**

No gene was found to correlate with response by RT–PCR. The microarray analysis identified a gene expression profile of 28 genes, with 12 upregulated in the pCR group and 16 upregulated in non-pCR. The leave-one-out cross-validation test exhibited 72% accuracy, 86% specificity, and 55% sensitivity. The 28-gene expression profile classified the 13 validation samples with 92% accuracy, 89% specificity, and 100% sensitivity.

**Conclusion:**

Our results suggest that genes not involved in classical cancer pathways such as apoptosis or DNA repair could be involved in responses to a trastuzumab–docetaxel-based regimen. They also describe for the first time a gene expression signature that predicts trastuzumab response.

Amplification and overexpression of human epidermal growth factor receptor 2 (HER2) is observed in 20–30% of invasive breast cancer (Slamon *et al*, 1987) and correlates with tumour progression and poor prognosis. Although the EGFR family stimulates mitogenesis through ligand-induced pathways, there is no known ligand for HER2. Increased HER2 expression induces a signalling pathway that involves Ras and Src, as well as PI3K/Akt, and is associated with tumour formation (Siegel *et al*, 1994).

Trastuzumab (Herceptin; F Hoffmann-La Roche, Basel, Switzerland) is a humanised monoclonal antibody directed against the HER2 protein. It produces significant (>50%) tumour regression in ∼15% of patients with HER2-positive metastatic breast cancer that is refractory to conventional therapy, and in ∼23% of patients when used as first-line therapy (Cobleigh *et al*, 1999). The addition of trastuzumab to standard chemotherapy significantly improves the response rate, response duration, and survival. The clinical benefits of trastuzumab-based therapies have been well documented in both adjuvant (Joensuu *et al*, 2006) and metastatic settings (Marty *et al*, 2005). However, the precise molecular pathways through which trastuzumab exerts its anti-tumour effects in breast cancer cells are not yet fully understood. Trastuzumab action involves multiple mechanisms, including the induction of apoptotic signalling pathways, cell cycle perturbation, and cellular cytotoxicity (Sliwkowski *et al*, 1999). Treatment with trastuzumab dephosphorylates and downregulates HER2, leading to significant clinical efficacy against HER2-positive breast cancer. It also sensitises breast cancer cells to chemotherapeutic agents, especially to tubulin-polymerising agents and radiation therapy (Baselga *et al*, 1998; Liang *et al*, 2003). It was shown that anti-HER2 monoclonal antibodies inhibit HER2-overexpressing breast cancer cells through G1 cell cycle arrest, which was associated with the induction of the cyclin-dependent kinase (CDK) inhibitor p27^kip1^ and reduction of CDK2 (Le *et al*, 2003). Trastuzumab may also inhibit the PI3K/Akt pathway by promoting PTEN activation (Nagata *et al*, 2004). Trastuzumab has been shown to reduce tumour volume and microvessel density in HER2-positive breast cancer models *in vivo* (Laughner *et al*, 2001; Izumi *et al*, 2002). Synergy with DNA-damaging drugs is thought to be due to trastuzumab-mediated inhibition of DNA repair. Trastuzumab partially inhibits the repair of DNA adducts *in vitro* after treatment with cisplatin and blocks unscheduled DNA synthesis after radiation (Pietras *et al*, 1994, 1999). Finally, trastuzumab has also been shown to be associated with immunoreactive actions through antibody-directed cellular cytotoxicity (ADCC) (Arnould *et al*, 2006).

Recently, trastuzumab-based neoadjuvant chemotherapy has been shown to achieve promising efficacy, with a good pathological complete response (pCR) rate, while being well tolerated in women with stage II or III HER2-positive breast cancer (Buzdar *et al*, 2005; Coudert *et al*, 2006, 2007). Among taxanes, docetaxel associated with trastuzumab shows evidence of improved efficacy in obtaining pCR rates.

In this study, we examined the expression of a panel of 30 genes involved in cell cycle progression, DNA repair, and apoptosis, which may have a putative role in trastuzumab resistance, in a series of breast carcinomas that had been treated with trastuzumab-based neoadjuvant chemotherapy. In parallel, we used microarray analysis on the same tumour samples to identify a potential marker of pCR that may have a prognostic value in identifying patients who are more likely to respond to trastuzumab therapy.

## Materials and methods

### Patients and samples

We retrospectively studied a population of 38 patients who had received trastuzumab in combination with chemotherapy as primary systemic therapy for their operable, HER2-positive, stage II/III breast cancer (Table 1). All patients provided written, informed consent for their tissue material and clinical data to be used for research purposes. Patients were treated in two open-label phase II clinical trials: TAXHER01 (*n*=29) and GETNA01 (*n*=9) (Coudert *et al*, 2006, 2007).

All patients received weekly neoadjuvant trastuzumab (4 mg kg^−1^ loading dose, followed by 2 mg kg^−1^ once weekly) in combination with either docetaxel alone (100 mg m^−2^ every 3 weeks for six cycles) or docetaxel (75 mg m^−2^ every 3 weeks for six cycles) combined with carboplatin (AUC 6) every 3 weeks for six cycles. The pCR rates were assessed using Chevallier's classification (Chevallier *et al*, 1993) 3 weeks after the last course of trastuzumab-containing neoadjuvant treatment. An absence of disease in the breast or in the lymph nodes, with or without *in situ* carcinoma, was considered to be a pCR. The HER2 status was determined using both immunohistochemistry and fluorescence *in situ* hybridisation (Coudert *et al*, 2006, 2007).

### HER-2 testing

The HER-2 status was analysed before treatment in each tumour according to ASCO guidelines for immunohistochemistry (IHC) or fluorescent *in situ* hybridisation (FISH) (Wolff *et al*, 2007) IHC was carried out with an anti-HER2 antibody (clone 4B5) on a Ventana Benchmark XT automate (Ventana Medical Systems, Tucson, AZ, USA). All tumours were considered as positive if >30% of tumour cells display a complete and strongly positive membrane staining. As described in a previous report (Coudert *et al*, 2006, 2007; Arnould *et al*, 2006), all biopsies were also retrospectively analysed with FISH procedures that confirm that all tumours included in this study displayed an *HER2* gene amplification with a mean of more than six copies of the *HER2* gene.

### RNA extraction

Needle core biopsy samples were taken at baseline, with one used for the initial diagnosis and two used for RNA extraction. All tissue samples were snap frozen and stored in liquid nitrogen, and only samples containing ⩾30% tumour cells were analysed further. Total RNA was extracted from tissue samples by using the TRIzol method as recommended by the manufacturer (Invitrogen Corporation, Carlsbad, CA, USA). The quantity, quality, and purity of extracted RNA were assessed using a NanoDrop 1000 spectrophotometer (NanoDrop, Wilmington, DE, USA) at 260 and 280 nm (the A_260/280_ ratio of pure RNA is higher than 1.8) and an Agilent 2100 bioanalyser (Agilent, Santa Clara, CA, USA). Total RNA from a pool of four normal mammary tissues was used as normal sample, and RNA extracted from the MCF-7 human breast cancer cell line was used to calibrate real-time quantitative and reverse transcriptase (RT)–PCR.

### RT–PCR and real-time quantitative PCR

One microgram of total RNA was reverse transcribed in 20 *μ*l of RT–PCR. Real-time quantitative PCR was carried out on an ABI PRISM 7300 (Applied Biosystems, Foster City, CA, USA) using the TaqMan method. Analysis of 18S ribosomal RNA was used to assess complementary DNA (cDNA) quality and as a reference control. Results were analysed at the Ct level and references for the genes analysed are summarised in Table 2. Survivin, caspase-3, and their splice variant expressions were determined by design primers and probes labelled at the 5′ end with FAM and at the 3′ end with TAMRA. Assays on Demand (Applied Biosystems) were used for the other studied genes. The results were analysed using either the 2^−ΔCt^ method for expression comparison or the 2^−ΔΔCt^ method (Vegran *et al*, 2007) for statistical analyses.

Statistical analyses were carried out with Statview 5.0 software (SAS Institute, Inc., Cary, NC, USA). The non-parametric Mann–Whitney *U*-test was used to compare gene expression with pathological response. Statistical significance was considered when *P*-value was <0.05.

### Microarray experiment

Microarray analyses were carried out using the Affymetrix-Microarray Platform of the Institute of Genetics and Molecular and Cellular Biology (IGBMC) and Génopole Alsace-Lorraine (Dr Philippe Kastner). The analysis used samples from 25 patients (11 with pCR and 14 with non-pCR) who constituted the training set. It was randomly constituted with the 25 first patients enrolled in the study. The resulting profile was validated using an independent and blinded group of 13 patients (four with pCR and nine with non-pCR) belonging to the test set. It was also randomly constituted with the 13 patients who joined the protocol after the beginning of training set microarray analysis.

The fluorescent nucleic acids hybridised onto the microarrays were prepared from total RNA. One microgram of total RNA was reverse transcribed into cDNA using a poly-dT with an extended region as a 3′ end primer. After second-strand synthesis, all the different double-strand cDNAs had a common 3′ end extension, which was used as a specific annealing site during PCR amplification. This unidirectional PCR amplification produced single-strand linear PCR products, which were labelled by random priming with dUTP-Cy5 (red) for the test samples or with dUTP-Cy3 (green) for the reference samples. Test and reference samples were co-hybridised onto microarrays. Human microarrays from the Affymetrix-Microarray Platform of the IGBMC and Génopole Alsace-Lorraine were used, onto which 25 000 genes were spotted. Reference genes were eliminated. Hybridised slides were scanned to detect fluorescence signals at high resolution. Fluorescent intensities were normalised and standardised using IGBMC in-house ‘Elea’ software, followed by a LOcal Weighted Estimates of Smooth Scatterplots (LOWESS) fitting-based method. Briefly, genes were selected as invariants from ranks of values in the Cy3 and Cy5 channels, and were then used in the LOWESS algorithm to compute the normalisation factor between the two channel values. This generated two values: the signal value *A*=Log_2_ (test value ^*^ reference value)/2 and the log ratio *M*=Log_2_ (test value/reference value).

### Microarray data analysis

Using *A* values, we determined the lowest median expression level of the population and excluded every gene with an *A* value lower than this. Using this heuristic filtering, we identified 14 829 genes for further analysis. From this subset of genes, statistical filtering was performed on *M* values using IGBMC in-house statistical ‘Zoe’ software. The Mann–Whitney *U-*test was then used with 1000 permutations to compare pCR and non-pCR rates. Only genes with *P*<0.002 were kept in the signature to discriminate pCR and non-pCR groups. *P*-values and *q*-values (for false discovery rate) were presented in Table 3.

The classification of patients constituting the test set was performed with the calculation of the correlation coefficient between the microarray values of each test patient and the mean of the 28-gene microarray expression value of pCR and non-pCR determined with the training set. A patient was classified as a pCR when the correlation coefficient obtained with the mean training pCR values was superior to the one obtained with the mean training non-pCR values, and inversely. As mentioned above, the classification of patients belonging to the test set was blindly performed. The comparison with real patient response was carried out later.

### Leave-one-out cross-validation test

The leave-one-out cross-validation test was performed on the training set patients. One patient was randomly suppressed. On the new 24-patient training set, a Mann–Whitney *U-*test was carried out with 1000 permutations to compare pCR and non-pCR rates. Only genes with *P*<0.002 were kept to generate a signature discriminating pCR and non-pCR groups. Thereafter, the excluded patient was classified with the calculation of the correlation coefficient between the microarray values of the patient and the mean of the gene microarray expression value of pCR and non-pCR determined with the 24-patient training set. One patient was excluded each time, generating 25 different tests.

## Results

### Analysis of selected gene expression by quantitative RT–PCR

When the relative expression of genes associated with cell cycle progression was compared with pathological response, it was found that the expression of these genes did not correlate with the observed pathological response. We next compared the relative expression of DNA repair genes with pathological response, and the results similarly showed that the expression of these genes did not correlate with pathological response. No relationship was found with the relative expression of apoptotic genes either.

### Microarray data analysis

**Figure 1:**
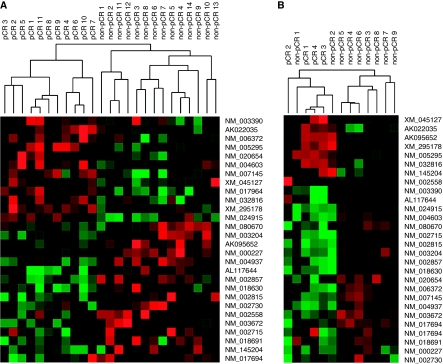
Hierarchical clustering of the 28 genes discriminating both pathological complete response (pCR) and non-pathological complete response (non-pCR) for the 25 patient training (**A**) and 13 patient test (**B**) sets. Green and red colours represent underexpression or overexpression centred to median array values, respectively.

Of the 25 patients in the training set, 11 (44%) showed pCR and 14 (56%) had non-pCR. Microarray analysis of tumour samples from these patients indicated that expression significantly differed between pCR and non-pCR tumour samples for 28 genes (Figure 1A). Among these 28 genes, 12 were more highly expressed in pCR tumour samples (WEE1, ZNF146, SENP7, GPR22, KIAA1549, SYNCRIP, SLC30A6, GRHL2, CCDC123, LOC340171, STX1A, cDNA FLJ11973 fis, and clone HEMBB1001221), and 16 genes were highly expressed in non-pCR samples (LOC158402, PITPNA, PPP2CA, SLC35A4, NFE2L1, C5orf3, PEX19, P2RX1, CDC14A, SENP8, PSMD11, CTNS, DER1, PRKACA, LAMA3, and FLJ20160) (Table 4). In addition, there was no difference observed for treatment effect (TAXHER01 or GETNA01) on this 28-gene expression profile.

The discriminatory 28-gene profile was validated using a leave-one-out cross-validation test. The analysis of the profile was carried out without previous knowledge of the patients' pathological response. One patient was excluded each time and was classified using a correlation coefficient based on the mean expression value of each selected gene for the pCR and non-pCR subsets. A patient was classified as being in the pCR group when their correlation coefficient was higher, with mean values above the non-pCR values, and vice versa. Using this approach, the leave-one-out cross-validation test classified 6 out of 11 pCR patients as having the pCR expression profile, and 12 out of 14 non-pCR patients into the non-pCR profile. Thus, the gene expression profile exhibited 55% sensitivity, 86% specificity, and 72% accuracy (Table 5).

To proceed further, the discriminatory 28-gene profile was then validated using the independent cohort of 13 patients. Analysis of the profile was carried out without earlier knowledge of the patients' pathological response. Each patient's tumour sample was classified using a correlation coefficient based on the mean expression value of each selected gene for the pCR and non-pCR subsets. A patient was classified as being in the pCR group when their correlation coefficient was higher, with mean values above the non-pCR values, and vice versa. Using this approach, our 28-gene profile correctly classified the four pCR patients as having the pCR expression profile, and 8 out of 9 non-pCR patients into the non-pCR profile (Figure 1B). Thus, our 28-gene profile for a trastuzumab–docetaxel-based regimen exhibited 100% sensibility, 89% specificity, and 92% accuracy (Table 6).

## Discussion

The aim of oncology is to provide the most appropriate cancer treatment to ensure the best patient response. However, it is very difficult to choose the best combination of chemotherapy agents, and it is necessary to develop new tools that will aid in making the best treatment choice. In this study, we explored gene expression profiles to predict response to trastuzumab–docetaxel-based chemotherapy in women with locally advanced HER2-positive breast cancer.

The real-time quantitative PCR study on 30 genes involved in cell cycle progression, DNA repair, or apoptosis revealed that these genes did not seem to be predictive for pathological response. Trastuzumab-induced apoptosis has been demonstrated in both breast tumour cell lines and breast carcinomas (Brodowicz *et al*, 2001; Milella *et al*, 2004; Emi *et al*, 2005; Henson *et al*, 2006). However, in our study, we failed to highlight a role for apoptosis-related genes in our response discriminating profile. This could be explained by immunoreactive actions through ADCC (Arnould *et al*, 2006).

Using microarray analysis, we generated a 28-gene profile that could discriminate between tumour samples that would attain a pCR and those that would not in response to treatment with a trastuzumab–docetaxel-based regimen. This profile was not affected by treatment effect (TAXHER01 or GETNA01), and the results confirm previous analyses that have commented on the association between pCR and HER2 amplification (Arnould *et al*, 2006; Coudert *et al*, 2006, 2007). In addition, the expression values of the 30 selected genes analysed with real-time quantitative PCR were concordant with those that overlapped with high-throughput microarray, confirming the absence of involvement of these genes.

In the leave-one-out cross-validation test, the classifier shows 72% accuracy, 86% specificity, and 55% sensitivity. The 28-gene expression profile classified the 13 test samples with 92% accuracy, 89% specificity, and 100% sensitivity. The performance with the test set is better than that with the training set, conforming previous observations showing that independent validation is the gold standard to evaluate the performance of the prediction rule (Michiels *et al*, 2007). However, the main characteristic of this classifier is high specificity, both with training and test sets, allowing the identification of patients resistant to trastuzumab–docetaxel-based treatment.

Among the genes identified in the profile, NFE2L1 was upregulated in the non-pCR group. NFE2L1 has been described to be a regulator of detoxifying enzyme expression, and, in association with Jun, is able to induce the expression of genes encoding detoxifying enzymes. As a result, overexpression of NFE2L1 could protect tumour cells by decreasing the toxicity of treatment. Moreover, overexpression of NFE2L1 was described as having the same impact as c-Myc overexpression (Morrish *et al*, 2003), suggesting that this gene could be implicated in resistance to chemotherapy.

Two small ubiquitin-like (SUMO)/sentrin-specific protease (SENP) family members were differentially expressed in both pCR and non-pCR groups. Thus, SENP7 was upregulated in the pCR group, whereas SENP8 was strongly expressed in the non-pCR group. Although the properties and targets of SENP7 have not yet been determined, it has been established that SENP8 is an NEDD8-, rather than SUMO-, specific protease (Hay, 2007).

The *WEE1* gene harbours a large expression level in the pCR group. This gene suppresses the activity of the Cyclin B1–Cdc2 complex, suggesting its implication in the response process to trastuzumab–docetaxel-based treatments (Yoshida *et al*, 2004). The *GRHL2* gene, which is overexpressed in the pCR group as well, is involved in the regulation of hTERT. The GRHL2 downregulation by siRNA induced a decrease in hTERT activity and increased the immortalisation process (Kang *et al*, 2009).

*PPP2CA* is described as an anti-apoptotic gene (Hu *et al*, 2009), which could explicate why it is overexpressed in the non-pCR group. Other genes are overexpressed in the non-pCR set. For example, CDC14A is able to interact with and inhibit p53 and the Cyclin B–Cdk1 complex (Paulsen *et al*, 2006), and PSMD11 has been found to be overexpressed in breast carcinomas (Deng *et al*, 2007).

Surprisingly, four genes that discriminated between responses to treatment have been described as being involved in either synaptic transmission (*SYNCRIP*, *P2RX1*, and *STX1A*) or brain development (*KLHL2*). These results could highlight a new role of these genes in breast cancer.

Several studies have analysed gene expression profiles of breast carcinomas treated with docetaxel-based regimens. A 92-gene profile was identified that discriminated between docetaxel-resistant and -sensitive breast carcinomas (Chang *et al*, 2003). The functional classes of these differentially expressed genes were apoptosis, cell adhesion or cytoskeleton, protein transport, signal transduction, RNA transcription, RNA splicing or transport, cell cycle, and protein translation. Further, a 512-gene signature was described as predicting pCR to primary systemic therapy with gemcitabine, epirubicin, and docetaxel (Thuerigen *et al*, 2006). This signature contained a predominance of genes encoding enzymes and proteins binding to nucleic acids, many of which were transcriptional regulators. Another study on 44 breast tumour tissues identified 85 genes that predicted a clinical response to docetaxel with 80% accuracy (Iwao-Koizumi *et al*, 2005). The most prominent characteristic in non-responders was the elevated expression of genes controlling the cellular redox environment (glutathione and thioredoxin systems). Lastly, an *in vitro* study recently identified 50 genes involved in docetaxel sensitivity that were able to predict the response in 22 out of 24 clinical samples that were used in Chang's study (Potti *et al*, 2006).

To date, only one study has used RNA profiling to predict responses to trastuzumab–vinorelbine-based treatments in patients with early HER2-positive breast cancer (Harris *et al*, 2007). In this study, resistant tumours exhibited a higher expression of several growth factors, growth factor receptors, the PI3K regulatory subunit p85, microtubule-associated protein 2, and some basal genes. Although the chemotherapeutic agent used with trastuzumab is different, this signature was not confirmed in an independent set of patients to validate the identified profile. In addition, no predictive genes were identified in pCR tumours.

In conclusion, our results suggest that genes not involved in classical cancer pathways, such as apoptosis, cell cycle progression, or DNA repair, could be involved in determining responses to a trastuzumab–docetaxel-based regimen. Importantly, our results identify for the first time a gene expression signature that predicts trastuzumab response in breast carcinoma. A consequence of individualised treatment is that it can be difficult to identify appropriate numbers of patients with similar characteristics who have been exposed to the same treatment regimen to adequately statistically power the study. Thus, the prognostic accuracy of the 28-gene profile that we identified will be confirmed in a new multi-centre cohort of patients using a multivariate analysis in a larger number of cases.

## Figures and Tables

**Table 1:** Demographic data

	**Training set (*n*=25)**	**Independent set (*n*=13)**	**Total (*n*=38)**
*Age (years)*			
⩽50	13	9	22
>50	12	4	16
			
*SBR grade*			
I	1	0	1
II	14	8	22
III	10	4	14
Unknown	0	1	1
			
*Hormone receptors*			
ER-negative	13	3	16
ER-positive	12	10	22
PR-negative	16	3	19
PR-positive	9	10	19
			
*Tumour size (cm)*			
<2	1	0	1
2–4	17	12	29
>4	6	1	7
ND	1	0	1
			
*Treatment*			
TH	18	11	29
TCH	7	2	9
			
*Pathological response*			
pCR	11	4	15
Non-pCR	14	9	23

Abbreviations: ER=oestrogen receptor; non-pCR=non-pathological complete response; ND=not determined; pCR=pathological complete response; PR=progesterone receptor; SBR=Scarff-Bloom-Richardson; TCH=trastuzumab+carboplatin+docetaxel; TH=trastuzumab+docetaxel.

**Table 2:** References and nucleotide sequences of primers and probes used in this study

**Function**	**Gene or transcript**	**Reference NCBI**	**Reference or sequences**
Cell cycle	cdc27	NM_001256	Hs01559427_m1
	SKP2	NM_032637	Hs01021867_m1
	p27	NM_004064	Hs00153277_m1
	p53	NM_000546	Hs00153340_m1
	c-Myc	NM_002467	Hs00153408_m1
	Cyclin B2	NM_004701	Hs00270424_m1
	RBX1	NM_014248	Hs00360274_m1
	CCL4	NM_002984	Hs99999148_m1
	CDC45l	NM_003504	Hs00907337_m1
			
DNA repair	XRCC2	NM_005431	Hs00538799_m1
	ERCC2	NM_000400	Hs00361161_m1
	MREIIA	NM_005591	Hs00967442_m1
	HMOX2	NM_002134	Hs01558390_m1
	MSH5	NM_002441	Hs00159268_m1
Apoptosis	Survivin	NM_001168	F: 5′-CCAGATGACGACCCCATAGAG-3′
			R: 5′-TTGTTGGTTTCCTTTGCAATTTT-3′
			P: 5′-CATTCGTCCGGTTGCGCTTTCC-3′
	Survivin-2B	NM_001012271	F: 5′-AAGAACTGGCCCTTCTTGGA-3′
			R: 5′-CCAAGTGCTGGTATTACAGGCGTA-3′
			P: 5′-ACTGCCCCACTGAGAACGAGCCA-3′
	Survivin-ΔEx3	NM_001012270	F: 5′-CCCAGTGTTTCTTCTGCTTCAA-3′
			R: 5′-TTCTTCGCAGTTTCCTCAAATTCT-3′
			P: 5′-ACGACCCCATGCAAAGGAAACCAACA-3′
	Survivin-3B	AB154416	F: 5′-CCAGATGACGACCCCATAGAG-3′
			R: 5′-AAGAACTGGCCCTTCTTGGA-3′
			P: 5′-CATTCGTCCGGTTGCGCTTTCC-3′
			F: 5′-GCTTTGTTTTGAACTGAGTTGTCAA-3′
	Survivin-2*α*		R: 5′-GCAATGAGGGTGGAAAGCA-3′
			P: 5′-AGATTTGAGTTGCAAAGACACTTAGTATGGGAGGG-3′
	Caspase-3	NM_032991	F: 5′-CTGGACTGTGGCATTGAGACA-3′
			R: 5′-AGTCGGCCTCCATGGTATTT-3′
			P: 5′-TGGTGTTGATGATGACATGGCGTGTC-3′
	Caspase-3s		F: 5′-AGAAGTCTAACTGGAAAACCCAAACT-3′
			R: 5′-CAAAGCGACTGGATGAACCA-3′
			P: 5′-ATTATTCAGGTTATTATTCTTGGCG-3′
	Casp8AP2	NM_012115	Hs00201640_m1
	Caspase-9	NM_032996	Hs00154261_m1
	ASC	NM_013258	Hs0154724_gH
	Fasl	NM_000639	Hs00899442_m1
	LTBR	NM_002342	Hs00158922_m1
	HSP90	NM_001040141	Hs00743767_sH
	TRAF5	NM_004619	Hs01072220_m1
	BCL-x	NM_001191	Hs00236329_m1
	CD40	NM_000074	Hs99999100_s1
			
Housekeeping	18S	x03205.1	Hs99999901_s1

Abbreviations: F=forward; NCBI=National Center for Biotechnology Information; P=probe; R=reverse.

**Table 3:** *P*-value and *q*-value for the 28 genes constituting the gene signature

**GenBank ID**	**Resampling *P*-value**	**q-value**
AK095652	<0.001	<0.001
NM_003390	0.001	0.001
AL117644	0.001	0.002
AK022035	<0.001	<0.001
NM_002715	0.001	0.004
NM_007145	0.001	0.007
NM_020654	<0.001	0.001
NM_080670	0.001	<0.001
XM_045127	<0.001	0.001
NM_003204	0.001	0.001
NM_005295	<0.001	<0.001
NM_006372	<0.001	<0.001
NM_018691	0.001	0.001
NM_002857	<0.001	<0.001
NM_002558	0.001	0.002
NM_017964	<0.001	<0.001
NM_003672	0.001	0.007
NM_024915	<0.001	<0.001
NM_145204	0.001	0.002
NM_002815	0.001	0.003
NM_004937	<0.001	<0.001
NM_018630	<0.001	<0.001
NM_032816	<0.001	0.002
NM_002730	0.001	<0.001
NM_000227	0.001	<0.001
NM_017694	0.001	0.001
XM_295178	<0.001	<0.001
NM_004603	0.001	0.001

**Table 4:** Details of the 28 genes contained in discriminating profile

**GenBank ID**	**UGCluster**	**Name**	**Symbol**	**GOabr**	**AgilentSpotID**
AK095652	Hs.494822	Hypothetical protein LOC158402	LOC158402		as00595
NM_003390	Hs.249441	WEE1 homologue (*Schizosaccharomyces* *pombe*)	WEE1	ATP binding, cytokinesis, mitosis, nucleus, protein amino-acid phosphorylation, protein serine/threonine kinase activity, protein tyrosine kinase activity, regulation of cell cycle, transferase activity	as02991
AL117644	Hs.429819	Phosphatidylinositol transfer protein, alpha	PITPNA	Intracellular, lipid binding, lipid metabolism, phosphatidylcholine transporter activity, phosphatidylinositol transporter activity, transport, visual perception	as03022
AK022035	Hs.659665	CDNA FLJ11973 fis, clone HEMBB1001221			as04760
NM_002715	Hs.483408	Protein phosphatase 2 (formerly 2A), catalytic subunit, alpha isoform	PPP2CA	RNA splicing, ceramide metabolism, cytosol, hydrolase activity, inactivation of MAPK, induction of apoptosis, manganese ion binding, membrane, microtubule cytoskeleton, mitochondrion, negative regulation of cell growth, negative regulation of tyrosine phosphorylation of Stat3 protein, nucleus, phosphoprotein phosphatase activity, protein amino-acid dephosphorylation, protein heterodimerisation activity, protein phosphatase type 2A complex, regulation of DNA replication, regulation of Wnt receptor signalling pathway, regulation of cell adhesion, regulation of cell cycle, regulation of cell cycle, regulation of cell differentiation, regulation of growth, regulation of transcription, regulation of translation, response to organic substance, second-messenger-mediated signalling, soluble fraction	as05104
NM_007145	Hs.643436	Zinc-finger protein 146	ZNF146	DNA binding, heparin binding, nucleus, regulation of transcription, DNA-dependent, zinc ion binding	as06024
NM_020654	Hs.529551	SUMO1/sentrin-specific peptidase 7	SENP7	Cysteine-type peptidase activity, nucleus, protein sumoylation, proteolysis and peptidolysis, ubiquitin cycle	as06108
NM_080670	Hs.406840	Solute carrier family 35, member A4	SLC35A4	Golgi membrane, carbohydrate transport, integral to membrane, sugar porter activity	as06408
XM_045127	Hs.605380	*Homo* *sapiens* KIAA1549 protein	KIAA1549		as06448
NM_003204	Hs.514284	Nuclear factor (erythroid-derived 2)-like 1	NFE2L1	DNA binding, haem biosynthesis, inflammatory response, morphogenesis, nucleus, regulation of transcription, DNA-dependent, transcription, transcription cofactor activity, transcription factor activity, transcription from RNA polymerase II promoter	as08524
NM_005295	Hs.657277	G protein-coupled receptor 22	GPR22	G-protein coupled receptor protein signalling pathway, integral to plasma membrane, receptor activity, rhodopsin-like receptor activity, signal transduction	as09257
NM_006372	Hs.571177	Synaptotagmin-binding, cytoplasmic RNA-interacting protein	SYNCRIP	RNA binding, RNA splicing, endoplasmic reticulum, nuclear mRNA splicing, through spliceosome, nucleotide binding, nucleus, ribonucleoprotein complex	as10291
NM_018691	Hs.166551	Chromosome 5 open reading frame 3	C5orf3		as11424
NM_002857	Hs.517232	Peroxisomal biogenesis factor 19	PEX19	Integral to membrane, membrane, peroxisomal membrane, peroxisome organisation and biogenesis	as11638
NM_002558	Hs.41735	Purinergic receptor P2X, ligand-gated ion channel, 1	P2RX1	ATP binding, ATP-gated cation channel activity, apoptosis, integral to plasma membrane, ion channel activity, ion transport, membrane, receptor activity, signal transduction, synaptic transmission	as12494
NM_017964	Hs.23248	Solute carrier family 30 (zinc transporter), member 6	SLC30A6	Cation transport, cation transporter activity, membrane	as15670
NM_003672	Hs.127411	CDC14 cell division cycle 14 homologue A (*Saccharomyces* *cerevisiae*)	CDC14A	Cell proliferation, cytokinesis, hydrolase activity, nucleus, protein amino-acid dephosphorylation, protein tyrosine phosphatase activity, protein tyrosine/serine/threonine phosphatase activity, regulation of cell cycle	as15820
NM_024915	Hs.661088	Grainyhead-like 2 (Drosophila)	GRHL2		as16749
NM_145204	Hs.513002	SUMO/sentrin-specific peptidase family member 8	SENP8	Cysteine-type peptidase activity, proteolysis and peptidolysis, ubiquitin cycle	as18913
NM_002815	Hs.655396	Proteasome (prosome, macropain) 26S subunit, non-ATPase, 11	PSMD11	Binding, proteasome complex (sensu Eukaryota)	as19535
NM_004937	Hs.187667	Cystinosis, nephropathic	CTNS	L-cysteine transport, L-cysteine transporter activity, amino-acid metabolism, integral to membrane, lysosomal membrane, transport	as20561
NM_018630	KIAA1549	Derlin 1	DERL1		as21050
NM_032816	Hs.599703	Coiled-coil domain containing 123	CCDC123		as21549
NM_002730	Hs.631630	Protein kinase, cAMP-dependent, catalytic, alpha	PRKACA	ATP binding, cAMP-dependent protein kinase activity, cAMP-dependent protein kinase complex, nucleus, protein amino-acid phosphorylation, protein serine/threonine kinase activity, transferase activity	as21885
NM_000227	Hs.436367	Laminin, alpha 3	LAMA3	Basement membrane, epidermis development, laminin-1, protein binding, receptor binding, regulation of cell adhesion, regulation of cell migration, regulation of embryonic development, structural molecule activity, structural molecule activity	as22719
NM_017694	Hs.644886	FLJ20160 protein	FLJ20160	Integral to membrane	as22767
XM_295178	XM_295178	Not available	LOC340171	LOC340171	as24280
NM_004603	Hs.647024	Syntaxin 1A (brain)	STX1A	Exocytosis, integral to membrane, intracellular protein transport, membrane, neurotransmitter transport, protein transporter activity, regulation of insulin secretion	as25304

**Table 5:** Performance of the 28-gene expression profile with the leave-one-out cross-validation test

	**Predicted**		
	**pCR**	**Non-pCR**	**Total**
*Observed*			
pCR	6	5	11
Non-pCR	2	12	14
Total	8	17	25
			
	**Cases**	**Percentage**	
Sensitivity	6/11	55	
Specificity	12/14	86	
Positive prediction value	6/8	75	
Negative prediction value	12/17	71	
Accuracy	18/25	72	

Abbreviations: Non-pCR=non-pathological complete response; pCR=pathological complete response.

**Table 6:** Performance of the 28-gene expression profile for the independent cohort response prediction

	**Predicted**		
	**pCR**	**Non-pCR**	**Total**
*Observed*			
pCR	4	0	4
Non-pCR	1	8	9
Total	5	8	13
			
	**Cases**	**Percentage**	
Sensitivity	4/4	100	
Specificity	8/9	89	
Positive prediction value	4/5	80	
Negative prediction value	8/8	100	
Accuracy	12/13	92	

Abbreviations: Non-pCR=non-pathological complete response; pCR=pathological complete response.
